# Swapping Birth and Death: Symmetries and Transformations in Phylodynamic Models

**DOI:** 10.1093/sysbio/syz039

**Published:** 2019-05-28

**Authors:** Tanja Stadler, Mike Steel

**Affiliations:** 1 Department for Biosystems Science and Engineering, ETH Zürich, Basel 4058, Switzerland; 2 Biomathematics Research Centre, University of Canterbury, Christchurch 4800, New Zealand

**Keywords:** Algebraic symmetries, Bayesian inference, birth–death models, maximum likelihood, phylodynamics, phylogenetics, speciation–extinction models

## Abstract

Stochastic birth–death models provide the foundation for studying and simulating evolutionary trees in phylodynamics. A curious feature of such models is that they exhibit fundamental symmetries when the birth and death rates are interchanged. In this article, we first provide intuitive reasons for these known transformational symmetries. We then show that these transformational symmetries (encoded in algebraic identities) are preserved even when individuals at the present are sampled with some probability. However, these extended symmetries require the death rate parameter to sometimes take a negative value. In the last part of this article, we describe the relevance of these transformations and their application to computational phylodynamics, particularly to maximum likelihood and Bayesian inference methods, as well as to model selection.

Linear birth–death models play a pivotal role in phylodynamics. These stochastic models provide a prior distribution on evolutionary trees (both the shape and edge length distribution) for Bayesian inference methods (Yang and Rannala 1997; Stadler et al. 2013). Moreover, these models allow biologists to estimate key parameters of macroevolution (such as speciation rates corresponding to birth rates and extinction rates corresponding to death rates) from reconstructed phylogenetic trees which were dated by fossil (or other time-sampled) evidence (Nee et al. 1994).

The study of such models dates back to some classical papers from the early to mid-20th century (Yule 1924; Kendall 1948a,b), and the application of these models to phylogenetics and phylodynamics flourished from the 1990s onwards (Nee et al. 1994; Rannala and Yang 1996). Further in-depth mathematical analysis (Aldous 2001; Maddison 2007; Aldous et al. 2009; Morlon et al. 2011; Lambert and Stadler 2013) has extended our understanding of the properties of these models and extensions that allow more complex processes of birth and death.

In this article, we identify and explore curious symmetries in fundamental birth–death model probability distributions when the birth and death rates ($\lambda$ and $\mu$) are swapped. This symmetry has been known in the case of complete sampling of individuals at present (Waugh 1958; Tavaré 2018), and we will start the article by providing an intuitive account of this symmetry that seems at first a little surprising. We extend this to the more general setting where a third parameter is introduced—the sampling probability $\rho$ of individuals sampled at the present—and show how analogous symmetries can be derived by a transformation that reduces these three parameters to just two ($\lambda', \mu'$). One can view these as “corrected” birth and death rates, except for the caveat that this new death rate $\mu'$ can now take negative values. A major advantage of working with the transformed pair of parameters ($\lambda', \mu'$) is that it captures the correct dimensionality of the process (namely 2), thereby avoiding the inherent redundancy present in the 3D parameterization that uses the triple $(\lambda, \mu, \rho)$. This viewpoint has implications for phylogenetic and phylodynamic inferences, both in the maximum likelihood and Bayesian settings, and we explore these implications in the latter part of our article.

## Birth–Death Symmetries

Consider a phylogenetic tree that evolves from a single ancestral individual according to a birth–death process, with a constant birth rate $\lambda \geq 0$ and a constant death rate $\mu\geq 0$. Suppose that at some time point in the tree, there are $n$ individuals present. Let $p_{n,m}(t\:|\:\lambda, \mu)$ be the probability that at time $t$ later, there will be $m$ individuals present. These transition probabilities are classical and provide a foundation for phylodynamic models. The starting point for this article is the following curious symmetry which goes back to (Waugh, 1958) and was recently highlighted again in (Tavaré, 2018):

(1) \begin{equation*}\label{ppeq} p_{1,1}(t\:|\:\lambda, \mu) = p_{1,1}(t\:|\:\mu, \lambda). \end{equation*}

This equation states the surprising result that the probability of one individual having one surviving descendant after time $t$ remains the same if we swap the birth rate ($\lambda$) and the death rate ($\mu$). Thus a process with a birth rate of, say, 100 and a death rate of, say, 1—a scenario with a very fast-growing population—has the same probability of having one surviving descendant as a process with a birth rate of 1 and a death rate of 100—a scenario where we know that the process eventually leads to extinction. This symmetry can be extended to more general scenarios, as stated in the following theorem.

Theorem 1. *For any non-negative value of $\lambda, \mu$ and any value of $n\geq 1$:*

$$p_{n,n}(t\:|\:\lambda, \mu) = p_{n,n}(t\:|\:\mu, \lambda).$$

*More generally, set $\tilde{p}_{n,m}(t|\lambda, \mu) := \lambda^n \mu^m p_{n,m}(t|\lambda, \mu).$ Then for all $m \geq 0$ and $n \geq 1$ the following birth–death interchange symmetry holds:*

$$\tilde{p}_{n,m}(t|\lambda, \mu) = \tilde{p}_{n,m}(t|\mu, \lambda).$$

This result has been established in Waugh (1958) and explicitly stated in Tavaré (2018) (an alternative formal proof of Theorem 1 is provided in the supplementary material available on Dryad at http://dx.doi.org/10.5061/dryad.57704ft). To provide some intuitive insight into this result, we now provide a direct and conceptually transparent Proof of Theorem 1 in the case where $n=m=1$ (i.e., equation (1)); the result for $n=m > 1$ follows by essentially applying the same idea. We start a birth–death process with one individual. The waiting time between “events” (a birth event or death event) is $\exp(n (\lambda+\mu))$, where $n$ is the number of individuals at the considered time point. Let $p=\frac{\lambda}{\lambda+\mu}$, and consider two different scenarios (one proceeds forward in time, the other backward):

- Scenario 1: The process starts at time 0 and is stopped at time $t>0$. At an event, with probability $p$, we add an individual and, with probability $1-p$. we remove an individual. Scenario 1 is a classic forward-in-time birth–death process.
- Scenario 2: The process starts at time $t>0$ and is stopped at time $0$. At an event, with probability $1-p$ we add an individual and, with probability $p$, we remove an individual. Scenario 2 is a birth–death process in reversed time with the birth and death rates being interchanged compared with Scenario 1.

Intuitively, the result of the time-reversed process with birth and death being interchanged is analogous to the forward-in-time process. However, we justify this intuition by a formal argument showing that the probability of observing one individual after time $t$ is the same under Scenario 1 and Scenario 2.

**Figure 1.**
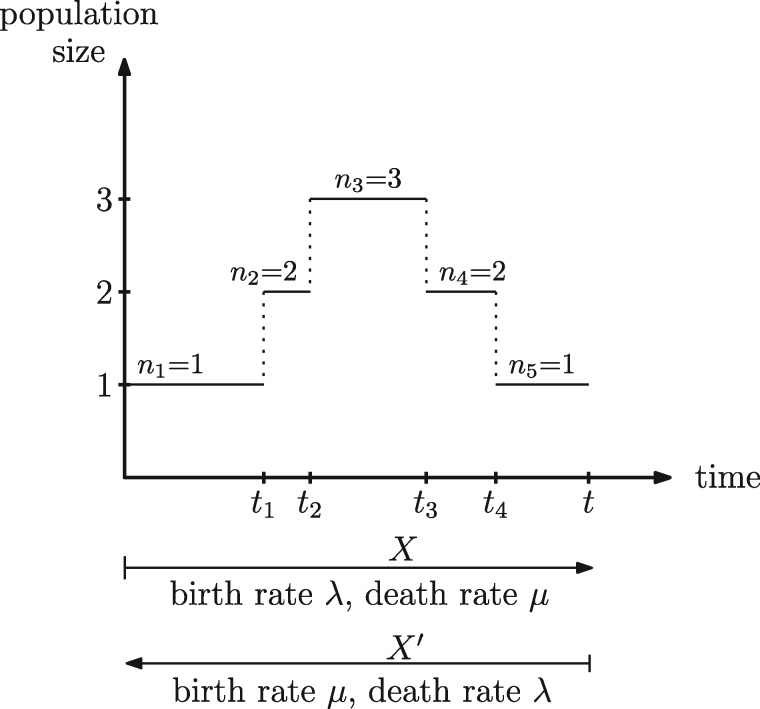
The forward-in-time birth–death process with realization $X$ and the equivalent time-reversed process with interchanged rates and realization $X'$.

Consider some population size trajectory $X$ that starts at time $0$ with one individual and ends with one individual after time $t$ (see Fig. 1 for an example). At each event, $X$ can grow or decrease by one. Let the number of growth events be $k$, which therefore also equals the number of death events. Denote the time of these $2k$ events by $t_1, t_2, \ldots t_{2k}$, and define $t_0=0$ and $t_{2k+1}=t$. See Figure 1 for an example with $k=2$.

The probability density of $X$ under Scenario 1, $L_1(X)$, is a product of the probability for the birth events, $p^k$, for the death events $(1-p)^k$, and the waiting times between events, $\prod_{i=1}^{2k} (\lambda + \mu) n_i e^{-(\lambda + \mu) n_i (t_i-t_{i-1})}$, where $n_i$ is the number of individuals prior to the event at time $t_i$. Finally, the term $e^{-(\lambda + \mu) (t-t_{2k})}$ stipulates that no subsequent event happens after the event at time $t_{2k}$. In summary, the probability density of $X$ under Scenario 1 for $k>0$ is:

\begin{align*}
L_1(X) & = p^k (1-p)^k (\lambda + \mu) e^{-(\lambda + \mu)((t_1-t_0) + (t_{2k+1}-t_{2k}))}\\
&\quad{} \prod_{i=2}^{2k} (\lambda + \mu) n_i e^{-(\lambda + \mu) n_i (t_i-t_{i-1})}.
\end{align*}

For $k=0$, we have

$$
L_1(X) = e^{-(\lambda + \mu) (t_{2k+1} - t_0)}.
$$

Now we reverse time in the realization $X$ and call it $X'$. Thus, $X'$ starts where $X$ ends, and $X'$ ends where $X$ starts. The probability density of $X'$ under Scenario 2 is then $L_2(X')$. We establish $L_2(X')$ analogous to the procedure above, with the birth events in $X$ being death events in $X'$ and vice versa. Thus, the same $p$ and $(1-p)$ factors are multiplied when calculating the probability density of $X'$ under Scenario 2, compared to the probability density of $X$ under Scenario 1. Furthermore, the waiting time contributions are the same for Scenario 1 and Scenario 2, and thus $L_1(X)=L_2(X')$.

Note that $p_{1,1}(t\:|\:\lambda,\mu)$ is the integral over all realizations $X$ under Scenario 1, $p_{1,1} (t\:|\:\lambda,\mu) = \sum_{k=0}^\infty \int_{\tau} L_1(X_{\tau, k}) d\tau$, where $X_{\tau, k}$ is a realization with $k$ birth events according to an event time vector $\tau = (t_1,t_2,\ldots, t_{2k})$.

Analogously, $p_{1,1} (t\:|\:\mu,\lambda) = \sum_{k=0}^\infty \int_{\tau} L_2(X'_{\tau, k}) d\tau$. Since $L_1(X_{\tau,k}) = L_2(X'_{\tau,k})$, each component in this integration has the same probability density and thus we have $p_{1,1} (t\:|\:\lambda,\mu)= p_{1,1} (t\:|\:\mu,\lambda)$.

One can directly extend this argument to establish Theorem 1 for any value of $n\geq 1$ by considering the associated forward-in-time and backward-in-time processes.

## General Symmetries under Incomplete Sampling

We continue to study a birth–death model with constant and non-negative birth and death rates $\lambda$ and $\mu$. However, we now allow each of the individuals present at time $t$ to be sampled (independently) with probability $\rho \in (0,1]$.

Let us first suppose that we start with one individual at time 0, and let $p_i(t\:|\:\lambda,\mu,\rho)$ be the probability that $i$ sampled descendants are observed (i.e., extant and sampled) at time $t$. The exact expressions for $p_i(t)=p_i(t\:|\: \lambda,\mu,\rho)$ are provided by the following theorem.

Theorem 2. *For $\lambda \neq \mu$, we have:*

$$p_n(t) =
\begin{cases}
1- \frac{\rho (\lambda-\mu)}{\rho \lambda + (\lambda(1-\rho)-\mu) e^{-(\lambda-\mu)t }}, & {\it{ if }}\,\, n=0;\\[8pt]
\frac{ \rho (\lambda-\mu)^2 e^{-(\lambda-\mu)t}}{( \rho \lambda + (\lambda(1-\rho)-\mu) e^{-(\lambda-\mu)t })^2 }, & {\it{ if }}\,\, n=1;\\[8pt]
p_{1,1} (t) (\lambda {q}(t))^{n-1}, & {\it{ if }}\,\, n>1;
\end{cases}
$$

*with*

$${q}(t):={q}(t\:|\:\lambda,\mu,\rho)=\frac{ \rho ( 1- e^{-(\lambda-\mu)t})}{ \lambda \rho + (\lambda(1-\rho)-\mu) e^{-(\lambda-\mu)t} }.$$

*For the critical case $\lambda=\mu$, we have:*

$$p_n(t) =
\begin{cases}
1- \frac{\rho}{1 + \rho \lambda t}, & {\it{ if }}\,\, n=0;\\[8pt]
\frac{\rho}{(1 + \rho \lambda t)^2}, & {\it{ if }}\,\, n=1;\\[8pt]
p_{1,1} (t) (\lambda {q}(t))^{n-1}, & {\it{ if }}\,\, n>1;
\end{cases}
$$

*with*

$${q}(t):={q}(t\:|\:\lambda=\mu,\rho)=\frac{ \rho t }{1+ \rho \lambda t}.$$

For $\lambda > \mu \geq 0$ and $\rho \in (0,1]$, the result is already provided in Stadler (2010), based on earlier work by Nee et al. (1994); Yang and Rannala (1997). The critical case for $\rho=1$ is provided for example in (Feller, 2008). For the proof of the remaining cases, refer to the Supplementary Material available on Dryad.

In what follows, we investigate the expressions for $p_i(t\:|\:\lambda,\mu,\rho)$ in detail, and identify symmetries with respect to adjusted birth and death rates.

### Negative “Death Rates” in the Case of Incomplete Sampling

We introduce two new variables $\lambda'$ and $\mu'$, which will play a key role in the remainder of the article. They are defined by $\lambda, \mu,$ and $\rho$ according to the following transformation:

$$\lambda' =\rho \lambda \mbox{ and } \mu' =\mu-\lambda(1-\rho).$$

Note that when $\rho=1$, we have $\lambda'=\lambda$ and $\mu'=\mu$. Further, for all vales of $\rho$ we have $\lambda'-\mu' = \lambda-\mu$ (thus $\lambda' \neq \mu'$ if and only if $\lambda \neq \mu$). Note also that $\mu'<0$ is entirely possible (e.g., when $\lambda=4 \mu$ and $\rho = 0.5$, we obtain $\mu'=-\mu$). In this case, $\mu'$ cannot easily be viewed as a death rate (nor as a birth rate); however, allowing $\mu'$ to take any real value (positive or negative) means that all parameter triplets $(\lambda,\mu,\rho)$ have a transformation to $(\lambda',\mu')$.

The following lemma is straightforward to verify using simple algebra (Stadler 2013).

Lemma 3. *For all $\lambda, \mu \geq 0,$ and $\rho \in (0,1]$, the four functions*

\begin{align*}
& \lambda {q}(t\:|\:\lambda,\mu,\rho), \mbox{ } \lambda (1-p_0(t\:|\:\lambda,\mu,\rho)),\\
&\quad{} \lambda p_{1,1} (t\:|\:\lambda,\mu,\rho), {\it{ and }}\,\, \lambda p_n(t\:|\:\lambda,\mu,\rho)
\end{align*}

*can be written as functions of only two parameters ($\lambda'$ and $\mu'$) when $\lambda \neq \mu$ (rather than the three parameters $\lambda, \mu, \rho$). When $\lambda=\mu$, these four functions can be written as functions of the single parameter $\lambda'$.*

In order to investigate symmetries, we define the following functions, which only depend on $\lambda'$, $\mu'$, and $t$ (rather than the four parameters $\lambda, \mu, \rho,$ and $t$) (this dependence on $\lambda'$, $\mu'$, and $t$ can easily be seen from Lemma 3). Let:

\begin{eqnarray*}
\tilde{p}_0(t\:|\: \lambda',\mu') &:=& \frac{1}{ \rho} (1-p_0(t\:|\:\lambda,\mu,\rho)), \\
\tilde{q}(t\:|\: \lambda',\mu') &:=& \frac{1}{ \rho} {q}(t\:|\:\lambda,\mu,\rho),\\
\tilde{p}_1(t\:|\: \lambda',\mu') &:=& \frac{1}{ \rho} p_{1,1} (t\:|\:\lambda,\mu,\rho),\\
\tilde{p}_n(t\:|\: \lambda',\mu') &:=& \tilde{p}_1(t\:|\: \lambda',\mu') {(\lambda' \tilde{q}(t\:|\: \lambda',\mu'))}^{n-1}.\\
\end{eqnarray*}

For $\lambda \neq \mu$, these equations are,

\begin{eqnarray*}
\tilde{p}_0(t\:|\: \lambda',\mu') &=& \frac{ \lambda'-\mu'}{ \lambda' - \mu' e^{-(\lambda'-\mu')t }}, \\
\tilde{p}_1(t\:|\: \lambda',\mu') &=& \frac{ (\lambda'-\mu' )^2 e^{-(\lambda'-\mu')t}}{( \lambda' -\mu' e^{-(\lambda'-\mu')t })^2 },\\
\tilde{p}_n(t\:|\: \lambda',\mu') &=& \frac{1}{ \rho} p_{1,1} (t\:|\:\lambda,\mu,\rho) (\lambda {q}(t\:|\:\lambda,\mu,\rho))^{n-1}\\
&=& \frac{1}{ \rho} p_n(t\:|\: \lambda,\mu,\rho),\\
\tilde{q}(t\:|\: \lambda',\mu') &=& \frac{ 1- e^{-(\lambda'-\mu')t}}{ \lambda' -\mu' e^{-(\lambda'-\mu')t}}.
\end{eqnarray*}

In particular, we have: $\tilde{p}_1(t\:|\: \lambda',\mu')= p_{1,1} (t\:|\:\lambda,\mu,\rho=1)$. This leads to the following symmetries with respect to $\lambda'$ and $\mu'$. A proof is provided in the Supplementary Material available on Dryad.

Theorem 4. *For $\mu' \geq 0$, the following symmetries hold:*

\begin{eqnarray*}
{\lambda' (1-\tilde{p}_0(t\:|\:\lambda', \mu'))} &=& { \mu' (1- \tilde{p}_0(t\:|\: \mu',\lambda'))},\\
\tilde{p}_0(t\:|\:\lambda', \mu') &=& \tilde{p}_0(t\:|\: \mu',\lambda')e^{(\lambda'-\mu')t},\\
\tilde{q}(t\:|\: \lambda',\mu') &=& \tilde{q}(t\:|\: \mu',\lambda'),
\end{eqnarray*}

*and for all $n \geq 1$:*

\begin{eqnarray*}
{(\mu')^{n-1}} \tilde{p}_n(t\:|\: \lambda',\mu') &=& {(\lambda')^{n-1}} \tilde{p}_n(t\:|\: \mu',\lambda').
\end{eqnarray*}

## Tree Probability Densities

**Figure 2.**
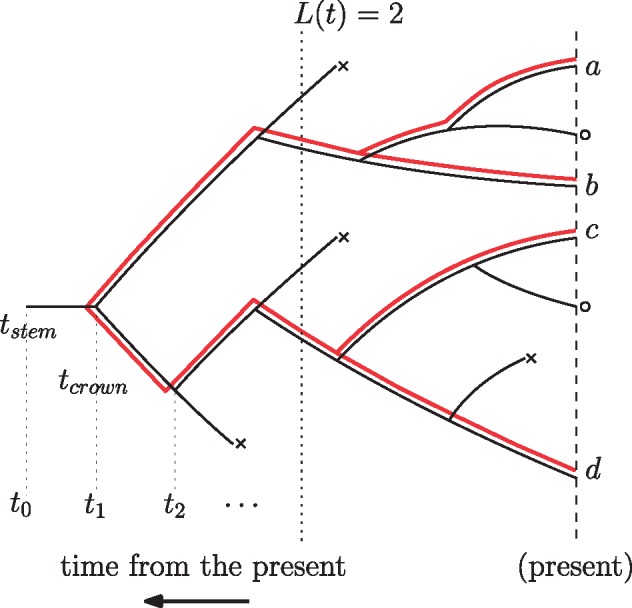
A phylogenetic tree ${\mathcal T}$ that evolves under a birth–death process with rates $\lambda, \mu$ and with sampling at the present with probability $\rho$. Lineages ending in a death (extinction) are marked by $\times$ whereas lineages at the present that are not sampled are marked by o. The reconstructed tree on the sampled extant individuals is indicated by the additinal lines starting at $t_1$.

Let ${\mathcal T}$ be a phylogenetic tree generated by a birth–death process starting with one individual and being stopped after time $t_0$. Each individual alive after time $t_0$ is sampled with probability $\rho$. In this tree, all extinct lineages are pruned, and only the lineages leading to the sampled tips are kept. Such a tree is also called the *reconstructed tree* (Nee et al. 1994), as indicated by the red lines in Figure 2. Let this tree have $n$ sampled tips and the branching times $t_1>t_2,\ldots>t_{n-1}$, where time is measured from the present time 0. Let $L(t)$ be the number of coexisting lineages of tree ${\mathcal T}$ at time $t$ (see Fig. 2).

Let $f({\mathcal T} \:|\: L(t_0)=1)$ be the probability density of the tree ${\mathcal T}$, and let $f({\mathcal T} \:|\: t_0 = t_{\rm stem})$ be the probability density of the tree ${\mathcal T}$, given that at least one individual is sampled at present. Thus $t_0$ is the stem age ($t_{\rm stem}$) of the process. For $\rho=1$, this corresponds to conditioning on nonextinction of the process. Let $f({\mathcal T} \:|\: t_0 = t_{\rm stem}, L_s(0)=n)$ denote the probability density of the tree ${\mathcal T}$, given that we sample exactly $n$ tips at present (denoted by $L_s(0)=n$).

The tree ${\mathcal T}$ in these formulations was a tree starting with one individual, leading to two lineages at time $t_1$ in the past. Alternatively, a tree ${\mathcal T}$ may start with two lineages at time $t_1$ ago; the probability of such a tree is $f({\mathcal T} \:|\: L(t_1)=2)$. Let $f({\mathcal T} \:|\: t_1=t_{\rm crown})$ be the probability density of the tree ${\mathcal T}$ conditioning on sampling at least one descendant individual from both initial lineages. Note that when conditioning on sampling, the time $t_1$ is the crown age of the clade ($t_{\rm crown}$). Furthermore, let $f({\mathcal T} \:|\: t_1=t_{\rm crown},L_s(0)=n)$ be the probability density of the tree ${\mathcal T}$ conditioned on sampling exactly $n$ tips at present. Finally, in the setting where $t_0$ is chosen uniformly at random from $(0,\infty)$, then a tree ${\mathcal T}$ conditioned on $n$ tips and integrated over all possible $t_0$ has probability density $f({\mathcal T}\:|\:L_s(0)=n)$.

In what follows, we assume $\lambda>0$ and thus $\lambda' > 0$; otherwise, we cannot obtain a tree with $n>1$.

Theorem 5. *The tree probability densities can be expressed as functions of $p_0(t\:|\:\lambda,\mu,\rho), p_{1,1} (t\:|\:\lambda,\mu,\rho)$ and ${q}(t\:|\:\lambda,\mu,\rho)$, or $\tilde{p}_0(t\:|\:\lambda',\mu'),\tilde{p}_1(t\:|\:\lambda',\mu')$ and $\tilde{q}(t\:|\:\lambda',\mu')$. Omitting the parameters $\lambda,\mu,\rho,\lambda'$, and $\mu'$ in these functions for easier reading, the expressions are given in the following table:*

**Table:** 

${\rm Tree probability densities}$	$(\lambda, \mu, \rho){\rm{\text{-}}parameters}$	$(\lambda', \mu'){\text{-}}{\rm parameters}$
${\rm Unconditioned}$		
$f({\mathcal T} \:|\: L(t_0)=1)$	$p_{1,1} (t_0) \prod_{i=1}^{n-1} \lambda p_{1,1} (t_i)$	$\rho \tilde{p}_1(t_0) \prod_{i=1}^{n-1} \lambda' \tilde{p}_1(t_i)$
$f({\mathcal T} \:|\: L(t_1)=2)$	$p_{1,1} (t_1)^2 \prod_{i=2}^{n-1} \lambda p_{1,1} (t_i)$	$\left( \rho \tilde{p}_1(t_0)\right)^2 \prod_{i=2}^{n-1} \lambda' \tilde{p}_1(t_i)$
${\rm Conditioned}$		
$f({\mathcal T} \:|\: t_0 = t_{\rm stem})$	$\frac{ p_{1,1} (t_0)}{1-p_0(t_0)} \prod_{i=1}^{n-1} \lambda p_{1,1} (t_i)$	$\frac{\tilde{p}_1(t_0)}{\tilde{p}_0(t_0)} \prod_{i=1}^{n-1} \lambda' \tilde{p}_1(t_i)$
$f({\mathcal T} \:|\: t_1=t_{\rm crown})$	$\left( \frac{ p_{1,1} (t_1)}{1-p_0(t_1)} \right)^2 \prod_{i=2}^{n-1} \lambda p_{1,1} (t_i)$	$\left(\frac{\tilde{p}_1(t_0)}{\tilde{p}_0(t_0)}\right)^2 \prod_{i=2}^{n-1} \lambda' \tilde{p}_1(t_i)$
$f({\mathcal T}\:|\:L_s(0)=n)$	$n \frac{ p_{1,1} (t_1)}{1- p_0(t_1)} \prod_{i=1}^{n-1} \lambda p_{1,1} (t_i)$	$n \frac{\tilde{p}_1(t_0)}{\tilde{p}_0(t_0)} \prod_{i=1}^{n-1} \lambda' \tilde{p}_1(t_i)$
$f({\mathcal T} \:|\: t_0 = t_{\rm stem},$		
$L_s(0)=n)$	$\prod_{i=1}^{n-1} \frac{ p_{1,1} (t_i)}{ {q}(t_0)}$	$\prod_{i=1}^{n-1} \frac{\tilde{p}_1(t_i)}{ \tilde{q}(t_0)}$
$f({\mathcal T} \:|\: t_1=t_{\rm crown},$		
$L_s(0)=n)$	$\frac{1}{(n-1)} \prod_{i=2}^{n-1} \frac{ p_{1,1} (t_i)}{ {q}(t_0)}$	$\frac{1}{(n-1)} \prod_{i=2}^{n-1} \frac{\tilde{p}_1(t_i)}{\tilde{q}(t_0)}$

We note that the expressions in the middle column have been presented in Stadler (2013) (equation 1–7), highlighting that $f({\mathcal T} \:|\: L(t_1)=2)$ goes back to Thompson (1975) for $\rho=1$, $f({\mathcal T} \:|\: t_1=t_{\rm crown})$ to Nee et al. (1994), and $f({\mathcal T} \:|\: t_1=t_{\rm crown},L_s(0)=n)$ to (Yang and Rannala, 1997) (both for $\rho \in (0,1]$). Furthermore, the probability density $f({\mathcal T} \:|\: t_0 = t_{\rm stem}, L_s(0)=n)$ for $\rho=1$ is described in Felsenstein (2004) and in earlier work by Rannala (1997). The idea of parameter transformation (right column) has been introduced for $f({\mathcal T}\:|\:L_s(0)=n)$ in (Stadler, 2009).

Remark 6. Only the expressions for the unconditioned tree probability densities (i.e., the equations not conditioning on observing at least one sample) depend on all three parameters $\lambda, \mu,$ and $\rho$. The remaining five expressions (the conditioned tree probability densities) only depend on two parameters ($\lambda', \mu'$), meaning only two out of the three birth–death parameters $\lambda,\mu,\rho$ can be inferred from the phylogenetic tree. This has already been observed for $f({\mathcal T}\:|\:L_s(0)=n)$ by (Stadler, 2009) and is then trivial to generalize for the other equations. Furthermore, based on Theorem 4, the expressions for $f({\mathcal T} \:|\: t_0 = t_{\rm stem}, L_s(0)=n)$ and $f({\mathcal T} \:|\: t_1=t_{\rm crown},L_s(0)=n)$ (i.e., the expressions where we condition on both the age of the process and the number of sampled tips) give the same result for $\lambda',\mu'$ and for when the parameters are swapped to $\mu',\lambda'$. For complete sampling, Rannala (1997) noticed this symmetry in $f({\mathcal T} \:|\: t_0 = t_{\rm stem}, L_s(0)=n)$ (this author also mentioned that this special symmetry had also been independently observed by Monty Slatkin). Note that $\mu' \leq 0$ is possible, whereas $\lambda' > 0$, thus the swapping is only well-defined if $\mu' > 0$.

## Implications for Empirical Data Analysis

### Tree Symmetries for Complete Sampling with Implications on Parameter Inference

As highlighted in Remark 6, we can, based on Corollary 1 of the supplementary material available on Dryad, directly conclude that

\begin{align*}
& f({\mathcal T} \:|\: t_0 = t_{\rm stem}, L_s(0)=n;\lambda,\mu) \\
& \quad{} = f({\mathcal T} \:|\: t_0 = t_{\rm stem}, L_s(0)=n;\mu,\lambda),\\
& f({\mathcal T} \:|\: t_1 = t_{\rm crown}, L_s(0)=n;\lambda,\mu )\\
& \quad{} = f({\mathcal T} \:|\: t_1 = t_{\rm crown}, L_s(0)=n;\mu,\lambda).
\end{align*}

Thus, we obtain the same probability density when swapping birth and death. As a consequence, we have to specify if the birth rate is bigger or smaller than the death rate prior to any analysis based on these equations.

### Mapping from $(\lambda',\mu')$ to the Birth–Death Model Parameters $(\lambda,\mu,\rho)$ with implications for Maximum Likelihood and Bayesian Inference

When using the tree probability densities in a maximum likelihood inference framework, the expressions are maximized over the parameters for a given tree. Based on the five conditioned tree probability density equations, we should optimize over $\lambda'$ and $\mu'$, with $\lambda' \in (0,\infty)$ and $\mu' \in (-\infty,\infty)$, instead of maximizing over the three parameters $\lambda, \mu,$ and $\rho$, as the latter parameterization induces a ridge in the likelihood surface and thus optimization is problematic. This is equivalent to optimizing when assuming complete sampling (and allowing the “death rate” $\mu'$ to be negative) and, in a second step, assuming a sampling probability $\rho$ and transforming from $(\lambda',\mu')$ to $(\lambda,\mu)$. This procedure was already suggested in (Stadler, 2009), Section 6.2 (up to pointing out the possibility for negative $\mu'$). We next investigate for which chosen values of $\rho$ we can transform $\lambda',\mu'$ to $\lambda,\mu$. A proof is provided in the Supplementary Material available on Dryad.

Theorem 7. *Let ${P}$ denote the conditioned tree probability density for an arbitrary tree $\mathcal{T}$ given $\lambda' \in (0,\infty)$ and $\mu' \in (-\infty,\infty)$. The expression for ${P}$ is given in the right column of Theorem 5. Each $(\lambda',\mu')$ has corresponding birth–death parameters $(\lambda \in (0,\infty), \mu \in [0,\infty), \rho \in (0,1])$, namely:*

- *Given $\mu'\geq 0$, we obtain the same tree probability density ${P}$ using the expression in the middle column of Theorem 5 with parameters $(\lambda=\lambda'/\rho, \mu = \mu'-\lambda'+\lambda'/\rho)$, where $\rho$ is any value in $\rho \in (0,1]$.*
- *Given $\mu'<0$, we obtain the same tree probability density ${P}$ using the expression in the middle column of Theorem 5 with parameters $(\lambda=\lambda'/\rho, \mu = \mu'-\lambda'+\lambda'/\rho)$, where $\rho$ is any value in $\rho \in (0,\frac{1}{1-\mu'/\lambda' }]$.*

In summary, given we estimate a negative $\mu'$, for some $\rho$, we cannot transform the parameters to $\lambda,\mu$. Thus, for parameter inference on empirical data, the best strategy might be to fix $\rho$ and then estimate $\lambda$ and $\mu$.

Given the dependency of $\lambda,\mu,$ and $\rho$ on only two parameters $\lambda'$ and $\mu'$, one may decide to perform a Bayesian analysis on $\lambda' \in (0,\infty),\mu' \in (-\infty,\infty)$ (see also Stadler (2009), Section 6.1). Care has to be taken though regarding the priors, since these priors play out in nonstraightforward ways. Assume, for example, that the analysis is performed by sampling $\lambda',\mu'$. For each sampled parameter pair, one might assume a $\rho \in (0,1]$ uniformly at random. Given that $\mu' \geq 0$, this would yield a uniform distribution on the chosen $\rho$. However, given that some sampled parameter pairs reveal $\mu'<0$, it follows that only a small $\rho$, namely $\rho \in (0,\frac{1}{1-\mu'/\lambda' }]$ is possible, meaning that overall, the samples on $\rho$ would be nonuniform, with a preference for small values of $\rho$. Thus, in the Bayesian setting, we need to assess the effective priors on $\lambda, \mu, \rho$ given the parameter nonidentifiability.

### Mappings between Birth–Death Model Parameters $(\lambda,\mu,\rho)$ and $(\hat{\lambda},\hat{\mu},\hat{\rho})$

Next, we characterize all birth–death parameters that are transformations of $\lambda, \mu, \rho$, the proof is again provided in the Supplementary Material available on Dryad.

Theorem 8. *Let $(\lambda,\mu,\rho)$ be birth–death parameters with the corresponding $(\lambda',\mu')$. There exist parameters $\hat{\lambda}>0,\hat{\mu} \geq 0,$ and $\hat{\rho} \in (0,1]$ with*

$$\lambda \rho = \hat{\lambda} \hat{\rho} = \lambda' {\it{ and }}\,\, \mu-\lambda(1-\rho) = \hat{\mu}-\hat{\lambda}(1-\hat{\rho}) = \mu'$$

*if $\mu / \lambda \geq 1$ (for all $\hat{\rho} \in (0,1]$) and if $\mu / \lambda < 1$ (for all $0<\hat{\rho} \leq \rho/(1-\frac{\mu}{\lambda})$).*

Note that the parameters $(\lambda,\mu,\rho)$ and $(\hat{\lambda},\hat{\mu},\hat{\rho})$ give thus rise to the same tree probability density.

Corollary 9. *With $\frac{\mu}{\lambda}<1$ (and thus $\hat{\rho} \leq \rho/(1-\frac{\mu}{\lambda})$) a transformation always exists for $\hat{\rho} < \rho$. However, a parameter transformation may not be possible for $\hat{\rho}>\rho$ (e.g., if $\frac{\mu}{\lambda} =0$, we cannot transform to ${\hat{\rho}}>\rho$).*

Next, we consider $\hat{\rho}=1$ (i.e., the transformation to the case of complete sampling). A further consequence of Theorem 8 is the following result from Stadler and Steel (2012).

Corollary 10. *With $\frac{\mu}{\lambda}<1$, a transformation exists to $(\hat{\lambda}>0,\hat{\mu} \geq 0,\hat{\rho}=1)$ if $\frac{\mu}{\lambda} \geq 1-\rho$. If $0 \leq \frac{\mu}{\lambda} < 1-\rho$, no transformation exists.*

#### Implications for proving properties of the birth–death tree distribution.

Properties of the birth–death tree distribution need to be known in order to test if empirical data are significantly different from these properties and thus the birth–death model has to be rejected for the given data. Sometimes, proofs of the properties of the conditioned tree distribution are carried out for complete sampling (i.e., for parameters $\hat{\lambda},\hat{\mu},\hat{\rho}=1$). Such properties also hold for incomplete sampling if $\frac{\mu}{\lambda} \geq 1$ or if $\frac{\mu}{\lambda} \geq 1-\rho$. To include the parameter space $0 \leq \frac{\mu}{\lambda} < 1-\rho$, the proof needs to be done with explicitly acknowledging incomplete sampling. This was noticed already in Stadler and Steel (2012).

#### Implications regarding model selection.

For a given phylogenetic tree, it is tempting to ask if a model with $\rho=1$ or $\rho=\hat{\rho}<1$ fits the data better. However, for every parameter combination $(\lambda,\mu,1)$, we also find a parameter combination $(\hat{\lambda},\hat{\mu},\hat{\rho})$ with both parameter triples having the same conditioned tree probability density. Moreover, there are parameter combinations $(\hat{\lambda},\hat{\mu},\hat{\rho})$ without a corresponding triplet where $\rho=1$ (see Corollary 9). Thus, the model with $\rho<1$ always gets more support than the model with $\rho=1$. In summary, such a test is meaningless because of the parameter nonidentifiability.

## Discussion

Birth–death models have been studied for almost 100 years (Yule 1924; Kendall 1948a). However, surprising properties are still being uncovered. Here, we presented some unexpected symmetries in birth–death models with incomplete sampling of individuals. In particular, a birth–death process with incomplete sampling can be described phylogenetically through two parameters instead of three parameters, resulting in parameter nonidentifiability.

Such parameter nonidentifiability has important consequences for using birth–death models in phylogenetic and phylodynamic inference. In particular, the likelihood surface of the three birth–death parameters $\lambda,\mu,$ and $\rho$ for a given tree has a ridge, and we can therefore only estimate two of the three parameters. Maximum likelihood estimation should thus be done for a fixed sampling probability. In Bayesian analysis, we need to carefully consider the effective prior when using such nonidentifiable parameter triplets.

Furthermore, we showed that for some of the parameter triplets ($\lambda,\mu,\rho$), their two-parameter description is, in fact, equivalent to a birth–death process with complete sampling. However, in some cases, the resulting ‘death’ rate is negative, and thus the transformed parameters cannot always be considered as a birth–death process with complete sampling. This means that we cannot simply prove properties of phylogenetic trees for complete sampling and then extrapolate to incomplete sampling, as we then miss some birth–death parameter combinations (namely the ones leading to a negative “death” rate). Furthermore, testing whether the data are completely sampled ($\rho=1$) or not ($\rho<1$) is not informative, as the models with $\rho<1$ always have more support: parameter triplets for incomplete sampling may only have corresponding complete sampling parameters with a negative “death” rate, whereas birth and death rates under complete sampling have a corresponding triplet for all $\rho \in (0,1]$.

The birth–death model presented here is the simplest model for speciation and extinction, or for transmission and recovery. However, it has limitations for explaining the data, as it assumes exponential growth of the population, although populations cannot have unlimited growth, and it assumes that all individuals are dynamically equivalent. There has been considerable work on extending the birth–death model to address such limitations (Maddison 2007; Morlon et al. 2011; Stadler 2011; Etienne et al. 2012; Stadler and Bonhoeffer 2013), but no symmetries and only very special parameter nonidentifiability has been observed (Stadler et al. 2013). It will be interesting to explore in the future whether the observed symmetries and nonidentifiabilities in our simple model are also present in these more complex models.

## Supplementary Material

Data available from the Dryad Digital Repository: http://dx.doi.org/10.5061/dryad.57704ft.
